# Production of *Camellia oleifera Abel* Seed Oil for Injection: Extraction, Analysis, Deacidification, Decolorization, and Deodorization

**DOI:** 10.3390/foods13101430

**Published:** 2024-05-07

**Authors:** Han Zhang, Mei Han, Xuejiao Nie, Xiaomeng Fu, Kunqiang Hong, Dongping He

**Affiliations:** 1 College of Food Science and Engineering, Key Laboratory for Deep Processing of Major Grain and Oil, Ministry of Education, Hubei Key Laboratory for Processing and Transformation of Agricultural Products, Key Laboratory of Edible Oil Quality and Safety for State Market Regulation, Wuhan Polytechnic University, 68 Xuefu South Road, Changqing Garden, Wuhan 430023, China; 2 School of Chemical Engineering and Technology, Tianjin University, Tianjin 300350, China

**Keywords:** camellia seed oil, injection oil, deacidification, decolorization, deodorization

## Abstract

Camellia seed oil (CSO), as a nutrient-rich edible oil, is widely used in foods, cosmetics, and other fields. In this work, the extraction, deacidification, decolorization, and deodorization processes of CSO were respectively optimized for meeting injectable oil standards. The results showed that the CSO extraction rate reached the highest level of 94% at optimized conditions (ultrasonic time, 31.2 min; reaction pH, 9.2; and reaction time, 3.5 h). The physicochemical indexes of CSO and 10 other vegetable oils were evaluated by the principal component analysis method, and the overall scores of vegetable oils were ranked as camellia seed oil > olive oil > rice oil > peanut oil > sesame oil > corn oil > soybean oil > sunflower oil > rapeseed oil > walnut oil > flaxseed oil. The physicochemical indicators of CSO were the most ideal among the 11 vegetable oils, which means that CSO is suitable as an injectable oil. Through the optimized processes of the deacidification, decolorization, and deodorization, the CSO acid value was reduced to 0.0515 mg KOH/g, the decolorization rate reached a maximum of 93.86%, and the OD_430_ was 0.015, meeting the requirement (≤0.045 of OD_430_) of injectable oil. After the deodorization process, these parameters of the refractive index, acid value, saponification value, iodine value, absorbance, unsaponifiable, moisture and volatiles, fatty acid composition, and heavy metal limits all met the pharmacopoeia standards of injectable oil in many countries and regions. The possibility of CSO as an injectable oil was first verified through refining-process optimization and nutritional index analysis, providing an important technical reference for the high-value utilization of vegetable oil.

## 1. Introduction

*Camellia oleifera Abel* is an important oil crop with a long history of cultivation and planting in China. Camellia seed oil (CSO) is also known as “oriental olive oil”, which is a relatively stable, natural, and healthy edible oil with a similar fatty acid composition to olive oil [1]. CSO is popular among consumers because of its attractive fatty acid composition [2], low iodine level [3], antioxidant properties [4], and rich content of natural active compounds [1]. Fewer oxidation products and trans-fatty acids are produced during high-heat food cooking process [5]. CSO has a unique aroma, and its fried food also exudes a lovely special flavor. The current industry study of CSO is mainly to meet people’s eating needs.

In addition, CSO is also widely applied in many fields, such as cosmetics and medicine. For example, it was reported that the glycosides of CSO were suitable for applications in cosmetic products due to their excellent antioxidant and moisturizing effects [6,7]. According to the record of the ancient Chinese Song Dynasty book Compendium of Materia Medica, CSO has the effect of softening and moisturizing hair, sterilizing, and relieving itching. Riangjanapatee et al. used Tween 80 and Varisoft 442 for CSO loaded nanostructured lipid carriers and developed a combination of both surfactants (NLC-C), which was an effective alternative in stimulating hair growth. Hair serum containing NLC-C obviously reduced sticky, oily, and greasy feeling [8]. In addition, CSO also contributed to the weight control and the improvement of cardiovascular-related risk factors in women with a high risk of cardiovascular disease [9]. CSO could effectively reduce the blood lipid level of obese mice host due to its fatty acid content [10]. Meanwhile, CSO could increase the activity of mice liver superoxide dismutase, thereby preventing excessive oxidation of lipids in cell membranes [11], as well as regulate the balance of Cl^−^ concentration in cardiomyocytes to prevent myocardial ischemia and reduce the incidence of arrhythmia [12].

Compared to edible oil or topical oil, injectable oil can provide essential fatty acids for some critically ill patients who cannot eat by mouth. Injectable oil was used as a carrier to improve the bioavailability of drugs and simultaneously make the drug release slowly and controllably for increasing the efficacy of drugs [13]. It was reported that edible vegetable oil was used as injectable oil through the refining process, such as corn oil [14], sunflower oil [15], soybean oil [16], fish oil [17], canola oil [18], peanut oil [19], olive oil [20], and brucea javanica oil [21]. These oils with relatively large production have a long research history and have been widely used in clinical treatment. In addition, zedoary turmeric oil [22], coix seed oil [23], sweet almond oil [24], monodispersed poppy seed oil [25], and croton oil [26], with relatively low production, were also reported as injectable oil. Among them, olive oil as an injectable oil has been widely used to treat short bowel syndrome [27], abnormal liver function, and essential fatty acid deficiency [28]. Studies have shown that curcumin-CSO emulsion was prepared by embedding curcumin from CSO. When the encapsulation rate was 83.53%, curcumin-CSO emulsion showed physical stability against environmental stresses, and CSO has the potential as a delivery system for lipophilic bioactive compounds [29]. It was shown that CSO was encapsulated into the hydrogel network via an emulsification process to synthesize a tough and antibacterial injectable hydrogel [30]. CSO shows great potential for medical applications, but there are still few relevant studies.

In recent years, the main extraction methods of CSO have included hot pressing extraction, cold pressing extraction, traditional solvent extraction, aqueous extraction, aqueous enzymatic extraction, and critical extraction [7]. Among them, the aqueous enzymatic extraction is recognized as a green, environmentally friendly, and sustainable oil extraction technology due to the advantages of no solvent residue, mild reaction conditions, and high recovery rate of residual oil [31]. In addition, due to the high content of peroxide substances and unsaturated fatty acids, and excessive acid value in crude oil, which can damage human health, the impure color cannot meet the injection standards. Therefore, the extraction, deacidification, decolorization and deodorization processes of CSO were respectively optimized to obtain injection CSO in this work. The physicochemical indicators of CSO and 10 other vegetable oils were comprehensively analyzed by principal component analysis to verify their possibilities as an injectable oil. This study can realize the pharmaceutical-grade injection use of CSO through refining processing, which is of great guiding significance for the development of high-value utilization of CSO and other vegetable oils.

## 2. Material and Methods

### 2.1. Experimental Material and Instruments

Camellia seed kernel was provided by a Hunan Runnong Ecological Tea Oil Co., Ltd. (Changsha, China), and the seeds were sieved using 40 mesh sieves. Cellulase and pectinase were purchased from Novozymes (China) Biotechnology Co., Ltd. (Tianjin, China). Neutral proteases and acid proteases were purchased from Shandong Anker Bioengineering Co., Ltd. (Heze, China). Alkaline protease was purchased from BIOSHARP (Benbu, China). Other chemicals and solvents were of analytical grade and purchased from Sinopharm Chemical Reagent Co., Ltd. (Shanghai, China). Soybean oil, rapeseed oil, sunflower oil, olive oil, peanut oil, and sesame oil were prepared by cold pressing. Walnut oil, flaxseed oil, corn oil, and rice oil were prepared by supercritical fluid extraction (SFE). For cold pressing, complete cold pressing oil was produced using a single-screw oil press (M222/20F, Miramar Nowa Wieś, Dziećmorowice, Poland), with the temperature of the generated oil tested being 45 ± 1 °C. For supercritical fluid extraction, SFE oil was obtained using an SFE Bio-Botanical Extraction System from Gaoke Pharmaceutical Equipment Co., Ltd. (GKSFE 120-50-05, Nantong, China). Specifically, 150 g of seed powder was placed in a 2 L extraction vessel, and carbon dioxide with a purity of 99.99% was injected into the SFE apparatus at a flow rate of 0.5 L/min under a pressure of 35 MPa for 3.5 min. The SFE process was carried out for 1 h at a temperature of 50 °C and pressure of 9 MPa. Subsequently, the extraction vessel was depressurized, and the oil was collected from the separating vessel.

### 2.2. Ultrasound-Assisted Aqueous Enzymatic Method

First, 100 g camellia seed powder (sifted by 40 meshes) was mixed with different volume (1:3~1:7 of material–liquid ratio) of distilled water. Then, the mixture was treated with an ultrasonic power of 300 W at 50 °C for 30 min. Following this, 2% (*w*/*w*) enzymes (cellulase, pectinase, acid proteases, neutral proteases, or alkaline proteases) were respectively added into the mixture. The temperature of the mixture was set at the corresponding reaction temperature (cellulase at 50 °C, pectinase at 50 °C, acid proteases at 45 °C, neutral proteases at 50 °C, or alkaline proteases at 50 °C) of the different enzymes, and 5 M HCl or 5 M NaOH was used to adjust its pH (cellulase at pH 5, pectinase at pH 3.5, acid proteases at pH 3, neutral proteases at pH 7, or alkaline proteases at pH 8.5). Following this, the temperature was set at 80 °C for 15 min, then lowered to 60 °C. The mixture was centrifuged at 5000 rpm for 25 min. The upper layer was crude CSO, the middle layer was emulsion and protein hydrolysate, and the lower layer was slag.

### 2.3. Deacidification of Camellia Seed Oil

The temperature of 100 g crude CSO was heated to 50 °C in a 250 mL conical flask, and then 9% (*w*/*v*) of NaOH was added. The temperature of mixture was the increased to 55, 65, 75, 85, or 90 °C, and the reaction time was 35, 45, 55, 65 or 70 min, respectively. Finally, the reaction solution was centrifuged at 6000 rpm for 15 min to obtain pre-deacidification CSO and insoluble matter. Following this, the pre-deacidification CSO was washed with an appropriate amount of water and centrifuged at 6000 rpm for 15 min to obtain deacidified CSO.

### 2.4. Decolorization of Camellia Seed Oil

First, 50 g of deacidified-CSO was put in a 250 mL flask and preheated at 65 °C for 5 min in an electrically heated thermostatic water bath, HWS-26 (Shanghai Qise Instrument Co., Ltd., Shanghai, China). The preheated CSO was mixed with 0.5 g of the decolorizer (activated carbon, attapulgite soil, diatomite, activated clay were purchased from Shanghai Shanpu Chemical Co., Ltd., Shanghai, China) at 65 °C for 30 min in the vacuum state (0.15 Pa). Following this, the temperature of the reaction solution was decreased to 30 °C, and the reaction solution was centrifuged at 4000 rpm for 10 min, obtaining decolorized CSO in the upper layer.

### 2.5. Deodorization of Camellia Seed Oil

The temperature of 50 g decolorized-CSO was increased to 200 °C in a vacuum machine, and then decolorized CSO was distilled at 200 °C with 1.5% (*w*/*w*) water vapor for 1 h. The temperature of distilled CSO was cooled down to room temperature, and the pressure of vacuum machine was restored to room pressure, generating deodorized CSO.

### 2.6. Analysis Method

Determination of fatty acid composition: In this process, 0.1 g oil sample was mixed with 1 mL of benzene-petroleum solvent (volume ratio of benzene: petroleum was 1:1), and 1 mL of 0.4 mol/L KOH-methanol solution was added into mixture. The mixture was incubated at room temperature for 10 min. Then, distilled water was added into stratify the oil sample. The upper organic layer sample was taken for chromatographic analysis. The chromatographic conditions were carried out according to the method of Ding et al. [32]. Column: Agilent SP-2560 (100 m × 25 μm, 0.2 μm); heating procedure, 100 °C for 4 min, 3 °C/min to 230 °C, hold for 20 min; carrier gas (N_2_) flow rate, 25 mL/min; pressure: 2.4 kPa; injection volume, 1 μL; shunt ratio, 15:1.

Oxidative stability analysis of oils: Rancimait oil oxidation stabilizer, type 892 was used to determine oil oxidation induction time. Then, 3.0 g of the oil sample and 60 mL of ultrapure water were added into the reaction tube and the absorber vessel of Rancimait oil oxidation stabilizer, respectively. The temperature was set to 120 °C, and the air flow was 20 L/h. The oxidation stability index (OSI) was automatically calculated by the instrument from the maximum second derivative of the conductivity curve.

### 2.7. Physicochemical Properties Analysis of Vegetable Oils

The peroxide value, being expressed in milli-equivalents of active oxygen of per kilogram oil, was calculated from the iodine which was released from potassium iodide. The value showed the evaluation criterion for incipient rancidity and indicated the conservation state of fatty matter. The method of peroxide value determination referred to AOCS Cd 8b-90. The iodine value was defined as the amount of iodine in grams calculated as the iodine absorbed by 100 g of the sample. The unsaturation of oils or fats was always measured by iodine value. The iodine value was determined based on the methods described by AOCS Cd 1c-85. The refractive indices of the oils were measured by an Abbe refractometer (WYA-2W, BELL Analytical Instruments (Dalian) Co., Ltd., Dalian, Chian.) at room temperature during the day and calibrated against pure water at 25 °C. The relative density, UV absorbance, acid values, saponification value, unsaponifiable value, moisture, and volatiles were detected according to the AOCS office methods Ea 7-95, Ea 9-56, Cd 5a-40, Cd 3-25, Ca 6a-40, and Ca 2d-25, respectively.

### 2.8. Data Analysis

The oil and protein extraction rate calculation formula is as follows:

(1) $$Oil\;extraction\;rate=\frac{\mathrm{Raw}\;\mathrm{material}\;\mathrm{oil}\;\mathrm{quality}\;-\;\mathrm{oil}\;\mathrm{quality}\;\mathrm{in}\;\mathrm{residue}}{\mathrm{raw}\;\mathrm{material}\;\mathrm{oil}\;\mathrm{quality}}\times 100$$

(2) $$Protein\;extraction\;rate(\%)=\frac{\mathrm{Amount}\;\mathrm{of}\;\mathrm{protein}\;\mathrm{in}\;\mathrm{raw}\;\mathrm{material}\;-\;\mathrm{amount}\;\mathrm{of}\;\mathrm{protein}\;\mathrm{in}\;\mathrm{residue}}{\mathrm{Amount}\;\mathrm{of}\;\mathrm{protein}\;\mathrm{in}\;\mathrm{raw}\;\mathrm{material}}\times 100$$

Determination of the decolorization rate

The absorbance of CSO was measured at a certain absorption wavelength, and the decolorization rate was calculated. The decolorization rate was calculated by the following formula:

(3) $$Decolorization=\frac{A1\;-\;A2}{A1}\times 100$$

A1: the absorbance before decolorization;

A2: the absorbance after decolorization.

All experiments were performed in triplicate, and the results are expressed as mean ± standard deviation (SD). Statistical analysis was conducted using Origin 2021. One-way analysis of variance (ANOVA) was performed on the data using SPSS Software (version number 19.0), and different letters were used to indicate significant differences (*p* < 0.05). In addition, SPSS 19 software was used to analyze the principal components of the 11 different vegetable oils, and finally, the cluster analysis was carried out.

## 3. Results and Discussion

### 3.1. Extraction of Camellia Seed Oil by the Ultrasound-Assisted Aqueous Enzymatic Method

**Figure 1:**
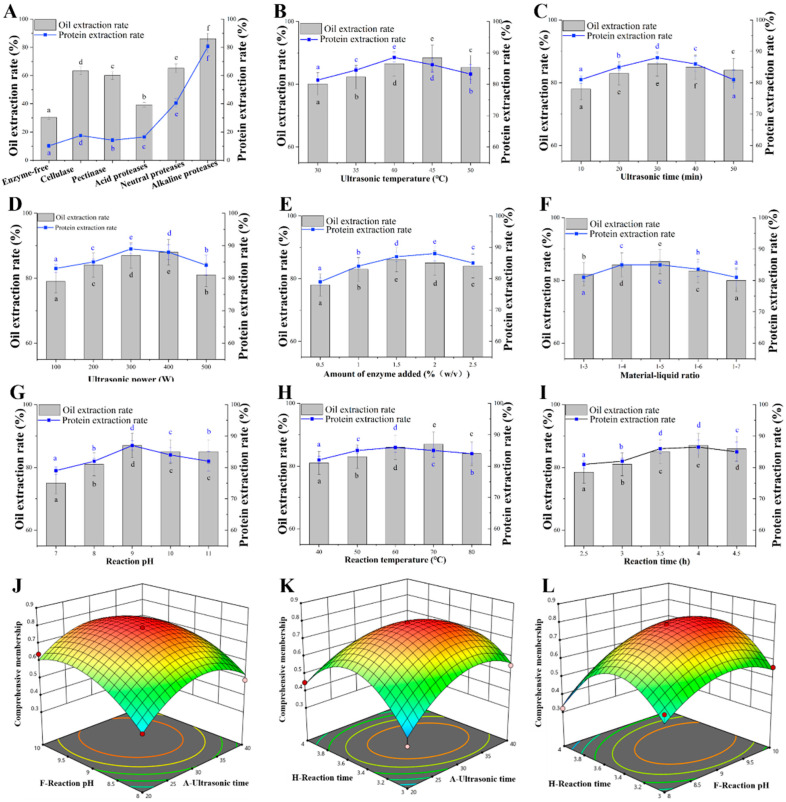
Extraction of camellia seed oil by the ultrasound-assisted aqueous enzymatic method. (**A**) Effect of the enzyme on the extraction of oil and protein. Ultrasonic time, 30 min; ultrasonic temperature, 35 °C; ultrasonic power, 300 W; material–liquid ratio, 1:5; amount of enzyme added, 2% (*m*/*m*); reaction time, 3 h. (**B**) Effect of the ultrasonic temperature on the extraction of oil and protein. (**C**) Effect of ultrasonic time on the extraction of oil and protein. (**D**) Effect of the ultrasonic power on the extraction of oil and protein. (**E**) Effect of the amount of enzyme added on the extraction of oil and protein. (**F**) Effect of the material–liquid ratio on the extraction of oil and protein. (**G**) Effect of the reaction pH on the extraction of oil and protein. (**H**) Effect of the reaction temperature on the extraction of oil and protein. (**I**) Effect of the reaction time on the extraction of oil and protein. (**J**) The response surface three-dimensional map of interaction by ultrasonic time and reaction pH. (**K**) The response surface three-dimensional map of interaction by ultrasonic time and reaction time. (**L**) The response surface three-dimensional map of interaction by pH and response time. Data are presented as the means and standard deviations of three independent experiments. Different lowercase letters indicate significant differences (*p* < 0.05).

The extraction rate of intracellular material can be enhanced by hydrolyzing cellulose backbone and pectin in cell walls [33]. Compared with the control method without enzymes, five enzymes of cellulase, pectinase, acid proteases, neutral proteases, and alkaline proteases assisted with the ultrasound could increase the extraction rate of CSO and protein (Figure 1A). When alkaline protease was used, the highest extraction rate of CSO and protein was 86% and 81%, 2.8-fold and 7.9-fold higher than that of control, respectively (Figure 1A). Therefore, alkaline protease was used to extract CSO and protein using the aqueous enzymatic method. Subsequently, the effects of the ultrasonic time, ultrasonic temperature, ultrasonic power, enzyme addition amount, material–liquid ratio, reaction pH, reaction temperature, and reaction time on the extraction rate were respectively tested. The extraction rate of SCO and protein was gradually increased with the increase of the ultrasonic temperature, ultrasonic time, and ultrasonic power but decreased at an exceeded set value (Figure 1B–D). The highest CSO extraction rate was 89% at a 45 °C ultrasonic temperature, and the highest protein extraction rate was 88% at a 40 °C ultrasonic temperature (Figure 1B). The protein extraction rate was decreased at a >40 °C ultrasonic temperature, while the oil extraction rate was gradually decreased at a >45 °C ultrasonic temperature (Figure 1B). This phenomenon might be caused by oil emulsification under the excessive ultrasonic temperature [34]. The extraction rate of CSO and protein achieved the maximum value, 86% and 88% at 30 min of ultrasonic time, and 89% and 89% at 300 W of ultrasonic power, respectively (Figure 1C,D). The dispersion and cavitation effect might be enhanced with the increase of ultrasonic power [35], resulting in the fragmentation of cell tissues or solid particles in the liquid, thereby increasing the oil extraction rate. In addition, lipoproteins or protein membranes formed during refining and surrounded by phospholipids and proteins in series were broken to release oil when protein was hydrolyzed by proteases, which was also the reason for the improvement of the oil–water dispersion [36]. 

As shown in Figure 1E, the extraction rate of CSO and protein was relatively ideal when the amount of enzyme added was 1.5~2%. Excessive water was not conducive to the enzymatic hydrolysis reaction, and appropriate material–liquid ratio was conducive to the enzymatic hydrolysis reaction (Figure 1F). Finally, single-factor experiment was respectively performed to examine the influence of reaction pH, reaction temperature, and reaction time on the extraction rate. These three factors exhibited almost identical effects on the extraction rate of CSO and protein, and the extraction rate was gradually increased in the appropriate range and gradually decreased in an exceeded value (Figure 1G–I).

Considering the cumbersome work of many factor optimization experiments, the Plackett-Burman method [37] was further used to quickly and effectively screen the main factors (Supplementary Materials Table S1). As shown in Supplementary Materials Table S2, the model’s corrected coefficient of determination R^2^ = 0.9672 proved that the experimental data could be explained by this model. Among the eight factors, ultrasonic time, reaction pH, and reaction time exhibited the most significant impact on the comprehensive membership degree (Supplementary Materials Table S2). Therefore, these three factors were further optimized with orthogonal experiment, and the other five factors were not significantly affected by the comprehensive membership degree (Supplementary Materials Table S2). The optimal conditions of the single-factor test were adopted: 45 °C ultrasonic temperature, 300 W ultrasonic power, 1:5 material–liquid ratio, 1.5% enzyme addition amount, and 60 °C reaction temperature.

Following this, Design Expert 8.0 software was used to design a three-factor three-level orthogonal test of the ultrasonic time, reaction pH, and reaction time (Supplementary Materials Table S3). A regression model was established, and the formula for the multiple quadratic regression equations for encoding the independent variables of the ultrasonic time, reaction pH. and reaction time was calculated as follows:

(4) $$Y1=-24.84850+0.20836\times A+2.43025\times F+6.51175\times H-7.275\times 10^{-3}\times AF+0.0181\times AH+0.0985\times FH\;-1.26\times 10^{-3}\times A2-0.13825\times F^{2}-0.99\times H^{2}$$

Y1—comprehensive degree of affiliation; A—ultrasound time (min); F—reaction pH; H—reaction time (h).

As shown in Table S4, the model difference was very significant (*p* < 0.01), while the misfit term was not significant (*p* > 0.05), and the adjusted coefficient of determination of the model R^2^ Adj = 0.9027, indicating that this regression equation could analyze and predict the experimental results. The influence of the interaction of two factors on the response value could be intuitively found from the three-dimensional diagram of the interaction between two factors in the response surface test (Figure 1J–L). The comprehensive degree of affiliation was gradually increased with the increase of ultrasonic time, reaction pH, and reaction time but decreased at an exceeded set value (Figure 1J–L). Design Expert 8.0 predicted the optimal process parameters were ultrasonic time, 31.2 min; reaction pH, 9.2; and reaction time, 3.5 h. The extraction rate of CSO and protein were consistent with the theoretical prediction value, reaching 94% and 91% under the predicted the optimal process.

### 3.2. Camellia Seed Oil Was Analyzed by Principal Component Analysis

**Figure 2:**
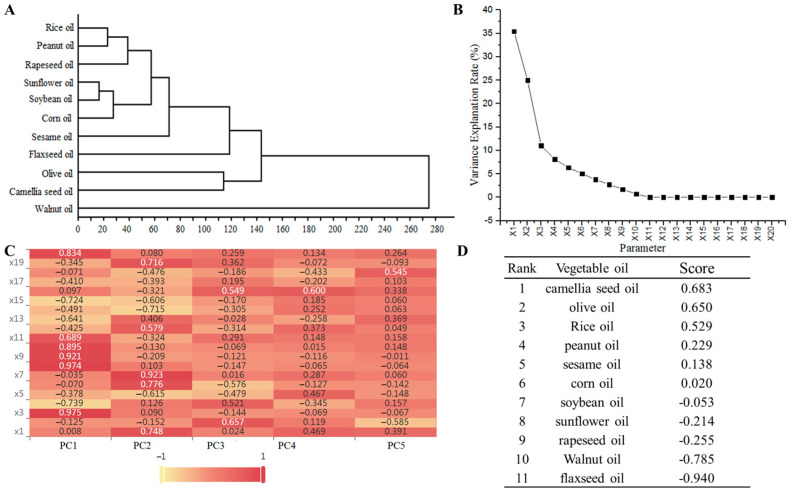
Physicochemical properties analysis of the 11 vegetable oils. (**A**) Clustering result of species identification of nine kinds of plants from Rosaceae. (**B**) Screen plot of principal component analysis. (**C**) Factor load matrix heat map. (**D**) Rank and overall scores of the 11 vegetable oils.

To verify the possibility of CSO as an injectable oil, different vegetable oils were systematically clustered and analyzed according to the physicochemical properties. Results showed CSO and olive oil were grouped together, which further confirmed CSO as “oriental olive oil” with a similar fatty acid composition and physicochemical properties to olive oil (Figure 2A and Supplementary Materials Table S5). Although the physicochemical properties of different vegetable oils were different, it still offers a variety of options for raw injectable oil. 

In addition, the 20 factors of different vegetable oils were analyzed by principal components to determine the characteristic values, so as to provide a scientific basis for the selection of vegetable oil for injection and the rational utilization and development of vegetable oil. As shown in Table S6 and Figure S1, there was a tight correlation between various factors, such as the positive correlation among palmitic acid, saturated fatty acid, and unsaponifiables, and the negative correlation between palmitic acid and refractive index. Palmitic acid and saturated fatty acid, saturated fatty acid and unsaponifiables, and unsaponifiables and water and volatiles showed positive correlations, which inicate information duplication between different factors. Therefore, principal component analysis (PCA) was used to select representative evaluation indicators to reduce information coverage. According to PCA results, the contribution of the first principal component (PC1) to the total variance was 35.44%, which reflected oil stability and performance. The better oil stability indicates better suitability as a raw for injection oil. The second principal component (PC2) and third principal component (PC3) contributed 24.99% and 11.03% to the total variance, mainly reflecting the unsaturation and saturation of oil, respectively (Table S7 and Figure 2B). Moreover, 8.15% of the fourth principal component (PC4) and 6.34% of the fifth principal component (PC5) contributed to the total variance. The cumulative variance contribution rate of the first five principal components reached 85.946% (Supplementary Materials Table S7 and Figure 2B), which could be a good description of the characteristics of different vegetable oil. By analyzing the principal component load factor (Supplementary Materials Table S8) and the heat map (Figure 2C), the importance of the hidden variables in each principal component can be analyzed. For example, the principal component load factors of X_3_, X_8_, X_9_, X_10_, X_11_, and X_20_ are higher in PC1, which means the stronger correlation between PC1 and these six factors. Meanwhile, the factor number was gradually decreasing from PC2 to PC5, with five factors (X_1_, X_6_, X_7_, X_12_, and X_19_) in PC2, two factors (X_2_ and X_16_) in PC3, one factor (X_16_) in PC4, and one factor (X_18_) in PC5, respectively (Supplementary Materials Table S8 and Figure 2C). These results further verified the ranked contribution rate of PC1 > PC2 > PC3 > PC4 > PC5. Following standardizing the initial factor component matrix (Supplementary Materials Table S9), the equation for each principal component was obtained as follows:

(5) $$F1=0.001X_{1}-0.0018X_{2}+0.137X_{3}-0.104X_{4}-0.053X_{5}-0.010X_{6}-0.005X_{7}+0.137X_{8}+\;0.130X_{9}+0.126X_{10}+0.097X_{11}-0.060X_{12}-0.091X_{13}-0.069X_{14}-0.102X_{15}+0.014X_{16}-\;0.058X_{17}-0.010X_{18}-0.049X_{19}+0.118X_{20}$$

(6) $$F2=0.150X_{1}-0.031X_{2}+0.018X_{3}+0.025X_{4}-0.025X_{5}-0.123X_{6}+0.156X_{7}+0.185X_{8}+0.021X_{9}\;-0.042X_{10}-0.026X_{11}-0.065X_{12}+0.116X_{13}+0.081X_{14}-0.143X_{15}-0.121X_{16}\;-0.064X_{17}-0.079X_{18}+0.143X_{19}+0.016X_{20}$$

(7) $$F3=0.010X_{1}+0.297X_{2}-0.065X_{3}+0.238X_{4}-0.218X_{5}-0.261X_{6}+0.007X_{7}-0.066X_{8}-0.054X_{9}\;-0.031X_{10}+0.132X_{11}-0.143X_{12}-0.011X_{13}-0.139X_{14}-0.078X_{15}+0.248X_{16}\;-0.088X_{17}-0.082X_{18}+0.164X_{19}+0.117X_{20}$$

(8) $$F4=0.288X_{1}+0.075X_{2}-0.043X_{3}-0.209X_{4}+0.285X_{5}-0.079X_{6}+0.177X_{7}-0.041X_{8}-0.072X_{9}\;+0.009X_{10}+0.09X_{11}+0.228X_{12}-0.158X_{13}+0.153X_{14}+0.113X_{15}+0.370X_{16}-0.124X_{17}\;-0.266X_{18}-0.048X_{19}+0.083X_{20}$$

(9) $$F5=0.308X_{1}-0.464X_{2}-0.052X_{3}+0.122X_{4}-0.115X_{5}-0.112X_{6}+0.047X_{7}-0.049X_{8}-0.007X_{9}\;+0.117X_{10}+0.126X_{11}+0.040X_{12}+0.291X_{13}+0.052X_{14}+0.049X_{15}+0.265X_{16}\;-0.083X_{17}+0.430X_{18}-0.069X_{19}+0.209X_{20}$$

According to the above formula, the comprehensive score formula was:

(10) $$F=0.412F_{1}+0.291F_{2}+0.128F_{3}+0.095F_{4}+0.074F_{5}$$

By substituting the original data into the above formula, scores and rankings for the different vegetable oils can be obtained for each of the principal components (Supplementary Materials Table S10). Finally, when the overall scores were calculated, the oils were ranked as camellia seed oil > olive oil > rice oil > peanut oil > sesame oil > corn oil > soybean oil > sunflower oil > rapeseed oil > walnut oil > flaxseed oil (Figure 2D). This ranking shows that the physicochemical indicators of CSO are the most ideal among the 11 vegetable oils, which means that CSO is suitable as an injectable oil. Although this ranking might be affected by other factors, such as the variety or origin of the raw material, this result still has important reference significance for the selection of injectable oil raw materials. The possibility of CSO as an injectable oil was proved through principal component analysis, but the physical indicators of crude CSO need to be further improved.

### 3.3. Camellia Seed Oil Deacidification

**Figure 3:**
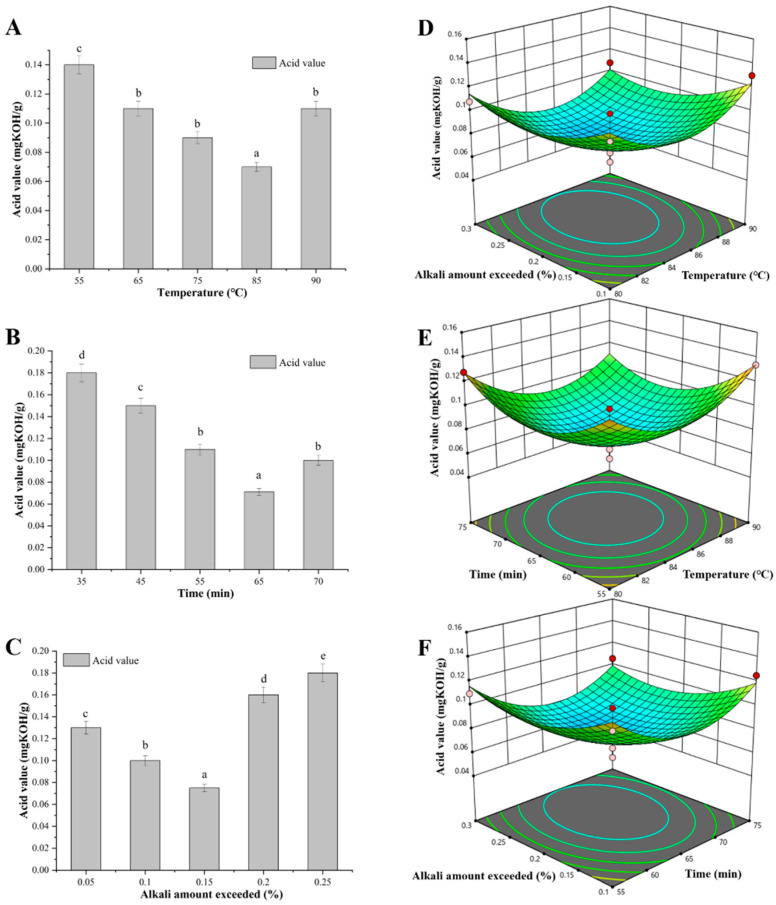
Deacidification of crude CSO. (**A**) Effect of the deacidification temperature on the acid value. Deacidification time, 3 5min; excess alkali amount, 0.10%. (**B**) Effect of the deacidification time on the acid value. Deacidification temperature, 50 °C; excess alkali amount, 0.10%. (**C**) Effect of excess alkali amount on the acid value. Deacidification temperature, 50 °C; deacidification time, 35 min. (**D**) The interaction of deacidification temperature and excess alkali amount on the acid value; (**E**) the interaction of deacidification temperature and time on the acid value; (**F**) the interaction of time and excess alkali amount on the acid value. Data are presented as the means and standard deviations of three independent experiments. Different lowercase letters indicate significant differences (*p* < 0.05).

The high levels of free fatty acids in crude CSO not only exhibit pungent odors but also lead to oxidative fission of oils, increasing the solubility of gel-soluble substances such as phospholipids, glycolipids, proteins, and fat-soluble oils, thereby reducing the quality of the oil [38]. Therefore, crude CSO with 3.06 mg KOH/g of acid value (Supplementary Materials Table S5) must be further deacidified. First, the effects of the deacidification temperature, deacidification time, and excess alkali amount on the deacidification of crude CSO were investigated, respectively. When the deacidification temperature was 85 °C, the acid value of CSO was the lowest level of 0.075 mg KOH/g, and then the value was increased with the increase of deacidification temperature (Figure 3A). The phenomenon might be caused by accelerated saponification rate of free fatty acids and enhanced hydrolysis of neutral oils at a high temperature [39]. With the increase of reaction time, the acid value of CSO tended to decrease significantly. When the reaction time was over 65 min, the acid value was increased (Figure 3B), indicating the probability of neutral oil being saponified and hydrolyzed changed with the change of contact time between CSO and lye. In addition, it is necessary to add an additional part of the alkali amount (excess alkali amount), which can prevent the reverse change of the reaction process and make up for the loss caused by the theoretical alkali amount in the physical and chemical reaction [32,39]. When the amount of excess alkali increased in the range of 0.05% to 0.15%, the acid value of CSO slowly decreased during the alkali refining process, but the acid value of CSO was gradually increased from 0.15% to 0.25% of the excess alkali (Figure 3C).

Following a three-factor, three-level orthogonal test of deacidification temperature, deacidification time, and excess alkali amount (Supplementary Materials Table S11), a regression model was established (Supplementary Materials Table S12), and the formula for the multiple quadratic regression equations for encoding the independent variables ultrasonic time, reaction pH, and reaction time was calculated as follows:

(11) $$Y2=2.30283-0.16388\times A-0.035288\times B-2.09750\times C-11.4753\times AB-0.010\times AC\;-15.2323\times BC+17.3588\times A^{2}+4.7551\times B^{2}+7.2883\times C^{2}$$

Y2—acid value (mg KOH/g); A—deacidification temperature (°C); B—reaction time (min); C—excess alkali amount (%).

As shown in Figure 3D–F, with the increase of deacidification temperature, reaction time, and excess alkali, the CSO acid value first decreased significantly and then increased flatly. After the above ANOVA and regression equation model, the optimized reaction condition parameters were determined by Design Expert 8.0 software: deacidification temperature, 80 °C; reaction time, 61.1 min; excess alkaline amount, 0.16%; and 0.0523 mg KOH/g of acid value was predicted. Under this condition, to perform a verification test, the actual acid value was 0.0515 mg KOH/g, meeting the requirement (below 0.1 mg KOH/g) of injectable oil.

### 3.4. Camellia Seed Oil Decolorization

**Figure 4:**
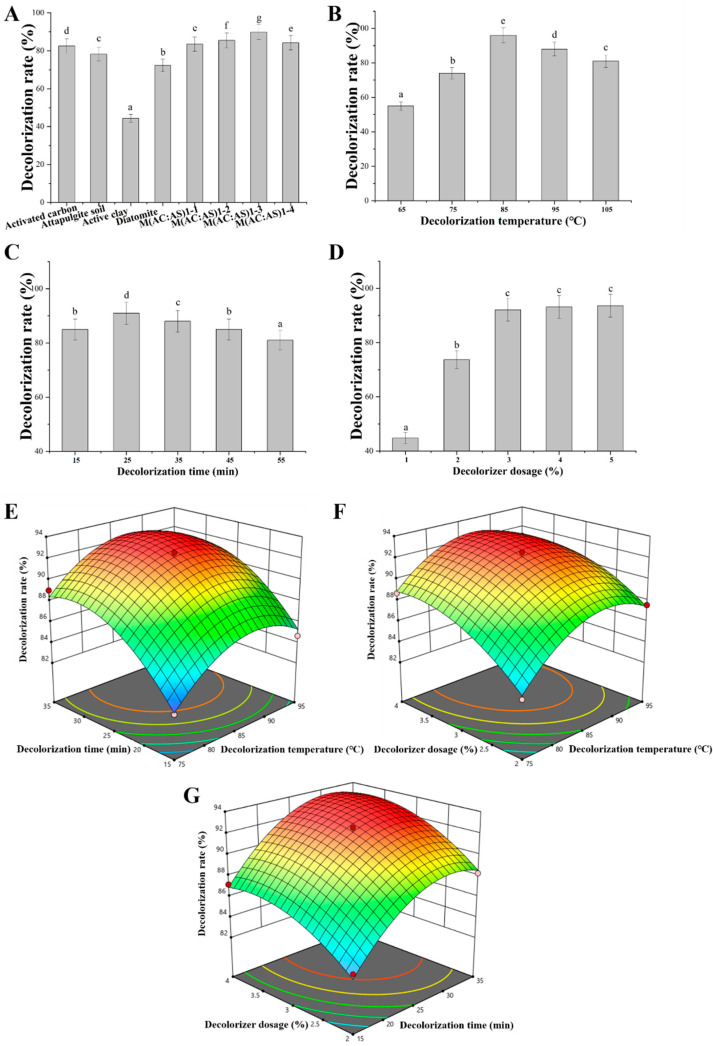
Decolorization of deacidified CSO. (**A**) The effect of different decolorizers on the decolorization rate. Decolorization temperature, 65 °C; decolorization time, 30 min; decolorizer dosage, 1.0% (*w*/*v*). (**B**) The effect of the decolorization temperature on the decolorization rate. Decolorization time, 30 min; mixed decolorizer dosage (AC:AS, mass ratio was 1:3), 1.0% (*w*/*v*). (**C**) The effect of the decolorization time on the decolorization rate. Decolorization temperature, 65 °C; mixed decolorizer dosage (AC:AS, mass ratio was 1:3), 1.0% (*w*/*v*). (**D**) The effect of the decolorizer dosage on the decolorization rate. Decolorization temperature, 65 °C; decolorization time, 30 min; mixed decolorizer dosage (AC:AS, mass ratio was 1:3). (**E**) The interaction of the decolorization temperature and decolorization time on the decolorization rate. (**F**) The interaction of the decolorization temperature and decolorizer dosage on the decolorization rate. (**G**) The interaction of the decolorization time and decolorizer dosage on the decolorization rate. Data are presented as the means and standard deviations of three independent experiments. Different lowercase letters indicate significant differences (*p* < 0.05).

The deacidification process alleviated the problem of darker CSO color, but it still could not meet injectable oil standard. Adsorption decolorization can not only remove some common pigments, but it can also remove the residual trace amount of soapstock, phospholipids and other gums, and polycyclic aromatic hydrocarbon chemicals formed in the process of deacidification in CSO [40]. To evaluate the decolorization rate, a UV/VIS spectrophotometer was used to determine the maximum absorption wavelength of CSO. Result showed that the maximum absorption wavelength of CSO was 430 nm (Supplementary Materials Figure S2). Subsequently, the decolorization effects of four decolorizers on CSO were evaluated. The decolorization effect of activated carbon was the highest of 82%. The decolorization effects of these four decolorizers were ranked as activated carbon > attapulgite soil > diatomite > activated clay (Figure 4A). To reduce the cost of the decolorization process, a mixture of activated carbon and attapulgite soil (AC:AS) was used as a decolorizer for decolorization. When the mass ratio of activated carbon and attapulgite soil was 1:3, the decolorization efficiency of grease was the highest 90% (Figure 4A). Then, the effects of the decolorization temperature, decolorization time, and AC:AS dosage on the decolorization effect of CSO were investigated by single-factor experiments. The gradual increase in decolorization temperature contributed to improving the decolorization effect of CSO, but the CSO may brown when the decolorization temperature was above 85 °C, resulting in a decrease in the decolorization effect (Figure 4B). The decolorization effect was the best when the decolorization time was 25 min. However, increasing the decolorization time may also accelerate the oxidation of the oil, which, in turn, would cause the color of the grease to darken again (Figure 4C). In addition, the decolorization rate was positively correlated with the dosage of AC:AS within a certain range, but when the dosage of AC:AS was in the range of 3~5%, the decolorization rate did not change significantly. Finally, a three-factor, three-level orthogonal test of decolorizer dosage, decolorization time, and decolorization temperature was implemented (Supplementary Materials Table S13); a regression model was established (Supplementary Materials Table S14); and the formula for the multiple quadratic regression equations for decolorizer dosage, decolorization time, and decolorization temperature was calculated as follows:

(12) $$Y3=-161.340+4.722\times A+1.948\times B+14.884\times C-7.689\times AB-0.046\times AC+0.013\times BC\;-0.027\times A^{2}-0.026\times B^{2}-1.586\times C^{2}$$

Y3—decolorization rate, A−decolorizer dosage, B−decolorization time, and C−decolorization temperature.

It was predicted that the maximum decolorization rate reached 93.66% when the decolorization temperature was 82 °C, the time was 33 min, and the dosage of the decolorizer was 3.65%. Under this condition, the actual decolorization rate was 93.86%, and the OD_430_ was 0.015, meeting the requirement (≤0.045 of OD_430_) of injectable oil.

### 3.5. Camellia Seed Oil Deodorization

After deacidification and decolorization, the acid value and color of CSO met the requirements of injection oil, but it the CSO still contained some low-molecular aldehydes, ketones, alcohols, and free fatty acids (FFAs). The deodorization process can remove peroxides and heat-sensitive pigments, as well as some polycyclic aromatic hydrocarbons and residual pesticides, and further improve the stability, color, and quality of the oil [41]. 

**Figure 5:**
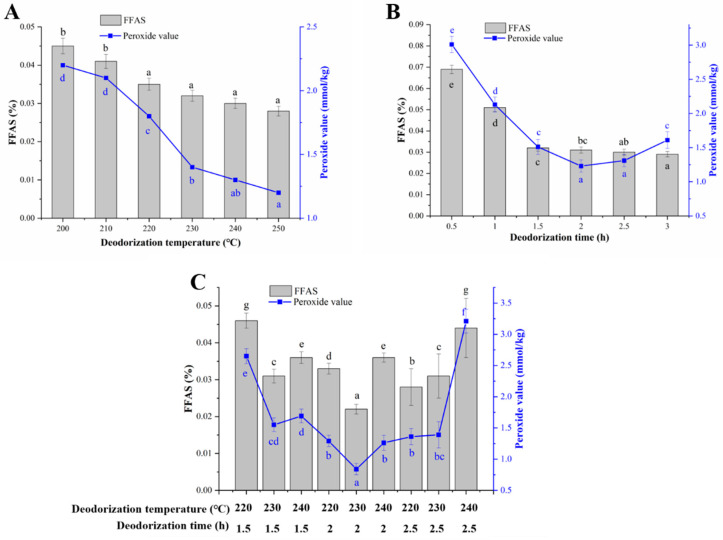
Deodorization of decolorized CSO. (**A**) Effect of deodorization temperature on deodorization. Vacuum degree, 0.096 MPa; deodorization time, 1 h. (**B**) Effect of deodorization time on deodorization. Vacuum degree, 0.096 MPa; deodorization temperature, 220 °C. (**C**) Orthogonal experimental of deodorization temperature and deodorization time. Data are presented as the means and standard deviations of three independent experiments. Different lowercase letters indicate significant differences (*p* < 0.05).

To further meet the standards of injection oil, the deodorization temperature and deodorization time of CSO were optimized based on the peroxide value and FFAs content. The vacuum degree of the vacuum deodorization device was set at 0.096 MPa, and the deodorization temperature was increased to 200–250 °C for 0.5–3 h, respectively. The FFAs level and peroxide value were decreased with the increase of temperature (Figure 5A). Their values were smoothly decreased when the temperature was above 230 °C. In addition, the FFAs level was not significantly changed when the deodorization time exceeded 1.5 h, and the peroxide value increased significantly after the deodorization time exceeded 2 h (Figure 5B). Therefore, its deodorization temperature was set at 220–240 °C, and the deodorization time was set to 1.5–2.5 h in orthogonal experiments. Orthogonal experimental results showed the best deodorization effect was achieved at 230 °C of deodorization temperature and 2 h of deodorization time. The FFAs level and peroxide value were decreased to the lowest level of 0.022% and 0.084 mmol/kg, respectively (Figure 5C). 

After the refining process, the characters, relative density, refractive index, acid value, saponification value, iodine value, absorbance, unsaponifiable, moisture and volatiles, fatty acid composition, and heavy metal limits were tested and analyzed (Table S15). Results showed these parameters all met the standard requirements of injectable oil (Table S15). In addition, the content of unsaturated fatty acids, palmitic acid, stearic acid, oleic acid, linoleic acid, and linolenic acid was increased to 90.49%, 7.12%, 2.03%, 81.09%, 9.12%, and 0.28%, respectively. It means the nutritional indicators of refined CSO also met the relevant requirements of injectable oil (Supplementary Materials Table S15). Finally, the effect of heating time and storage time on the color of refined CSO was determined. Refined CSO was stored at room temperature for 150 days or heated at 105 °C for 4 h. Its absorbance value was only slightly increased and still met the relevant requirement of injectable oil (Supplementary Materials Figure S3 and Table S15). These results also indicate that refined the CSO still had excellent stability after heating or storing for a long time.

## 4. Conclusions

In this study, CSO was extracted by an ultrasound-assisted aqueous enzymatic method, and the extraction rate of CSO reached the highest level after optimizing extraction conditions. The fatty acid composition and conventional physicochemical indexes of CSO and 10 other vegetable oils were comprehensively evaluated and analyzed by the principal component analysis method, indicating that CSO is also suitable as raw material for injection oil. The refined processes of deacidification, decolorization, and deodorization were carried out to make CSO an excellent raw material for injection oil. The results of this study provide useful information for the development of injectable CSO, but other harmful factors and nutritional components must be considered in future studies.

## Data Availability

The original contributions presented in the study are included in the article and Supplementary Materials, further inquiries can be directed to the corresponding author.

## Supplementary Materials

The following supporting information can be downloaded at: https://www.mdpi.com/article/10.3390/foods13101430/s1, Figure S1: Correlation coefficient heat map; Figure S2: Absorption wavelength of CSO; Figure S3: The effect of heating time (A) and storage time (B) on the color of refined CSO; Table S1: Plackett-Burman test for oil extraction and protein extraction ^a^; Table S2: Variance and significance analysis of Plackett-Burman test; Table S3: Experimental design and results of Box-Benhnken in oil extraction and protein extraction; Table S4: Variance analysis of parameters in regression equation of integrated membership grade in oil extraction and protein extraction; Table S5: The physicochemical indexes of 11 vegetable oils; Table S6: The correlate matrix between the factors ^a^; Table S7: Total variance interpretation; Table S8: Factor load factor; Table S9: Feature vector standardization; Table S10: Principal components and overall scores; Table S11: Experimental design and results of Box-Benhnken in CSO deacidification; Table S12: Variance analysis of parameters in regression equation of integrated membership grade in CSO deacidification; Table S13: Experimental design and results of Box-Benhnken in CSO decolorization; Table S14: Variance analysis of parameters in regression equation of integrated membership grade in CSO decolorization; Table S15: The fatty acid composition and conventional physicochemical indexes of CSO and other pharmacopoeias.
