# Supplementary material for: Production of Camellia oleifera Abel Seed Oil for Injection: Extraction, Analysis, Deacidification, Decolorization, and Deodorization
Source: Foods. 2024 May 7;13(10):1430. doi: 10.3390/foods13101430 (PMC11120317; doi:10.3390/foods13101430)
Supplement: Supplementary file 1 [file foods-13-01430-s001.zip › foods-2978813-supplementary.pdf]

Production of *Camellia oleifera Abel* seed oil for injection: Extraction, Analysis, Deacidification, Decolorization and Deodorization

Zhang Han[1], Han Mei[1], Nie Xue-Jiao[1], Fu Xiao-Meng[12], Hong Kun-Qiang[1]*, He Dong-Ping[1]

[1]Key Laboratory of Edible Oil Quality and Safety for State Market Regulation, Hubei Key Laboratory for Processing and Transformation of Agricultural Products, College of Food Science and Engineering, Wuhan Polytechnic University, 68 Xuefu South Road, Changqing Garden, Wuhan, 430023, P. R. CHINA

[2]School of Chemical Engineering and Technology, Tianjin University, Tianjin 300350, P.R. CHINA

* Corresponding author: Kun-Qiang Hong

E-mail: hongkq@whpu.edu.cn; Phone: +86-13237172182;

Address: Wuhan Polytechnic University, 68 Xuefu South Road, Changqing Garden, Wuhan, 430023, P. R. CHINA

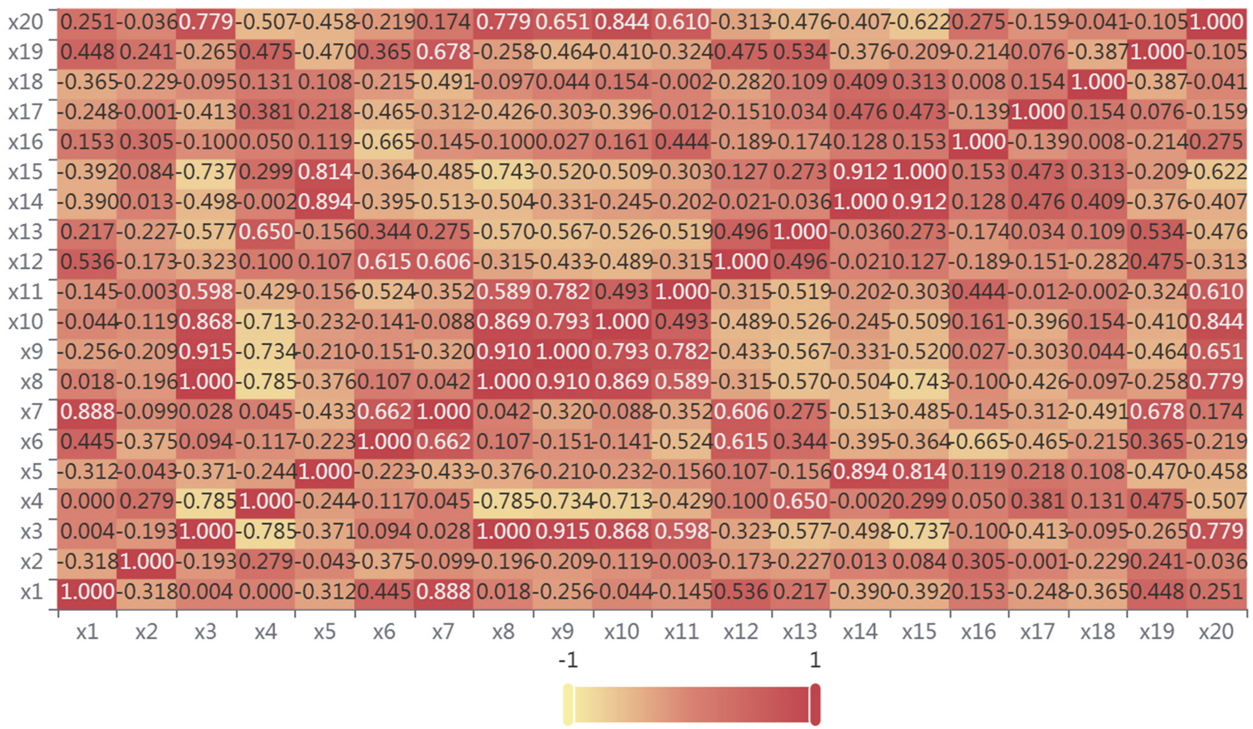

Figure S1 Correlation coefficient heat map

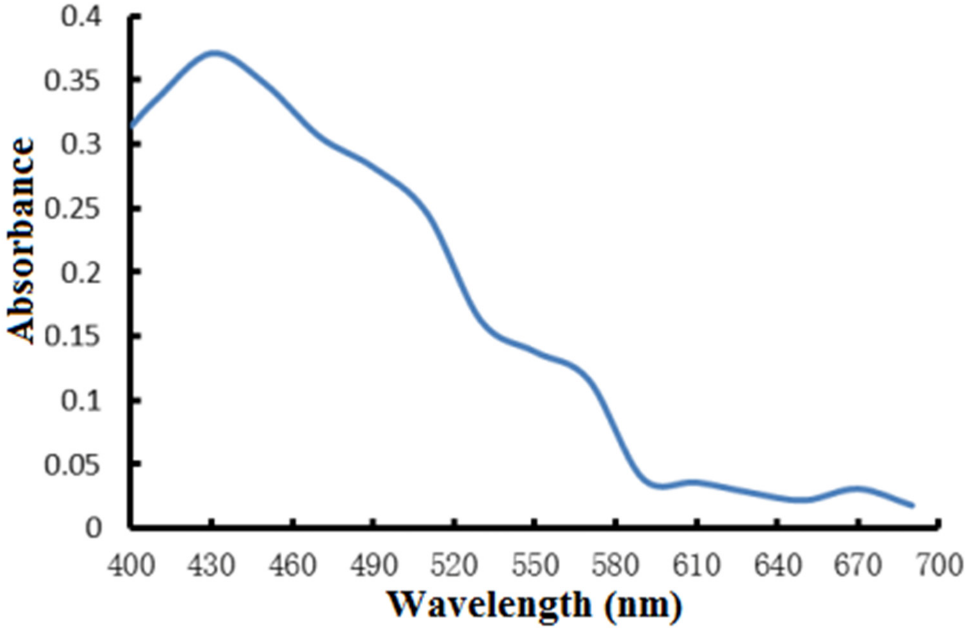

Figure S2 Absorption wavelength of CSO.

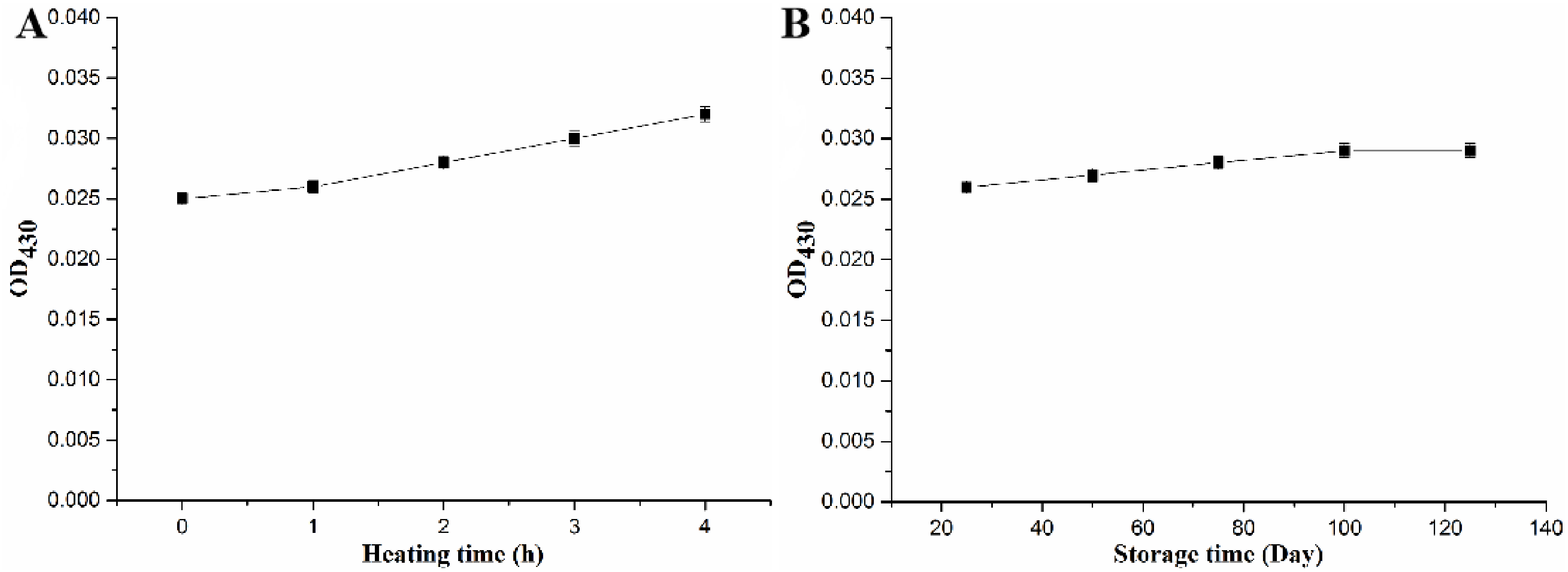

Figure S3 The effect of heating time (A) and storage time (B) on the color of refined CSO.

Supplemental material Table S1 Plackett-Burman test for oil extraction and protein extraction[a]

NO.	Ultrasonic temperature	Ultrasonic time	Ultrasonic power	Material-liquid ratio	Amount of enzyme added	Reaction pH	Reaction temperature	Reaction time	Oil extraction rate(%)	Protein extraction rate(%)	Oil extraction rate membership	Protein extraction rate membership	Comprehensive membership
1	-1	-1	1	-1	1	1	-1	1	86.68	90.25	0.278	0.686	0.482
2	-1	-1	-1	1	-1	1	1	-1	86.23	89.1	0.201	0.47	0.335
3	1	1	-1	1	1	1	-1	-1	87.23	87.07	0.385	0.088	0.236
4	-1	1	1	1	-1	-1	-1	1	90.44	88.86	1	0.424	0.712
5	-1	-1	-1	-1	-1	-1	-1	-1	88.84	86.61	0.692	0	0.346
6	1	-1	1	1	-1	1	1	1	87.93	87.55	0.519	0.178	0.348
7	1	-1	1	1	1	-1	-1	-1	87.78	88.19	0.49	0.298	0.394
8	-1	1	1	-1	1	1	1	-1	85.22	89.71	0	0.584	0.292
9	1	1	1	-1	-1	-1	1	-1	87.23	88.47	0.384	0.35	0.367
10	1	-1	-1	-1	1	-1	1	1	88.73	87.90	0.672	0.244	0.458
11	-1	1	-1	1	1	-1	1	1	87.22	91.92	0.382	1	0.691
12	1	1	-1	-1	-1	1	-1	1	87.65	88.23	0.465	0.305	0.385

[a]A-Ultrasonic time; B-Ultrasonic temperature; C-Ultrasonic power; D-Material-liquid ratio; E-Amount of enzyme added; F-Reaction pH; G-Reaction temperature; H-Reaction time

$$\text{Membership} = \frac{\text{target values} - \text{Minimum target value}}{\text{Maximum target value} - \text{Minimum target value}}$$

Comprehensive membership = 0.5×Oil extraction rate membership ＋ 0.5×Protein extraction rate membership

Supplemental material Table S2 Variance and significance analysis of Plackett-Burman test

Source of variance	Sum of squares	DF	Mean squared	F-value	P-value	Significance
Model	0.228672	8	0.028584	10.51785	0.0394	*
A-Ultrasonic time	0.037408	1	0.037408	13.76487	0.034	*
B-Ultrasonic temperature	0.008533	1	0.008533	3.139948	0.1754	
C-Ultrasonic power	0.001728	1	0.001728	0.63584	0.4835	
D-Material-liquid ratio	0.012416	1	0.012416	4.568748	0.1221	
E-Amount of enzyme added	0.003	1	0.0003	0.110389	0.7616	
F-Reaction pH	0.066008	1	0.066008	24.28861	0.0116	*
G-Reaction temperature	0.000341	1	0.000341	0.125598	0.7465	
H-Reaction time	0.101936	1	0.101936	37.50877	0.0088	**
Residual	0.008153	3	0.002718			
Cor Total	0.236825	11				
R-Squared	0.9672					
Adj R-Squared	0.9082					
$Y=0.0258-5.58E^{-3}\times A+5.333E^{-3}*B+2.4E^{-4}\times C+0.0643\times D+1E^{-2}\times E-0.0742\times F-5.33E^{-4}\times G+0.18433\times H$						

Supplemental material Table S3 Experimental design and results of Box-Benhnken in oil extraction and protein extraction

NO.	A-Ultrasonic time[a]	F-Reaction pH[b]	H-Reaction time[c]	Oil extraction rate(%)	Protein extraction rate(%)	Oil extraction rate membership	Protein extraction rate membership	Comprehensive membership
1	-1	-1	0	85.63	90.01	0.18	0.65	0.42
2	-1	1	0	86.75	92.11	0.33	0.94	0.64
3	0	1	-1	87.69	90.11	0.46	0.67	0.56
4	0	-1	1	88.95	85.23	0.63	0.00	0.32
5	-1	0	-1	88.75	85.69	0.61	0.06	0.33
6	-1	0	1	87.96	88.12	0.50	0.39	0.45
7	0	0	0	90.21	90.89	0.81	0.77	0.79
8	0	0	0	90.12	90.98	0.79	0.79	0.79
9	1	-1	0	87.56	89.23	0.44	0.55	0.49
10	1	0	-1	86.21	91.36	0.26	0.84	0.55
11	0	-1	-1	84.32	92.55	0.00	1.00	0.50
12	0	1	1	88.01	88.56	0.50	0.45	0.48
13	1	0	1	84.52	91.01	0.03	0.79	0.41
14	0	0	0	91.63	92.01	1.00	0.93	0.96
15	1	1	0	90.32	88.30	0.82	0.42	0.62

[a]Ultrasonic time was set to 20 (-1), 30 (0) and 40 (1) min;

[b]Reaction pH was set to 8 (-1), 9 (0) and 10 (1);

[c]Reaction time was set to 3(-1), 3.5(0) and 4(1)h.

$$\text{Membership} = \frac{\text{target values} - \text{Minimum target value}}{\text{Maximum target value} - \text{Minimum target value}}$$

Comprehensive membership = 0.5×Oil extraction rate membership ＋ 0.5×Protein extraction rate membership

Supplemental material Table S4 Variance analysis of parameters in regression equation of integrated membership grade in oil extraction and protein extraction

Source	Sum of Squares	df	Mean Square	Value	p-value	Significance
Model	0.33	9	0.037	16.17	0.0034	**
A-A	6.61E-03	1	6.61E-03	2.89	0.1497	
F-F	0.041	1	0.041	17.77	0.0084	*
H-H	9.80E-03	1	9.80E-03	4.29	0.0931	*
AF	2.03E-03	1	2.03E-03	0.89	0.3897	
AH	0.017	1	0.017	7.4	0.0418	*
FH	2.50E-03	1	2.50E-03	1.09	0.3435	
A^2	0.071	1	0.071	31.11	0.0026	**
F^2	0.044	1	0.044	19.11	0.0072	**
H^2	0.17	1	0.17	75.57	0.0003	**
Residual	0.011	5	2.29E-03			
Lack of Fit	0.011	3	3.81E-03			
Pure Error	0	2	0			
Cor Total	0.34	14				
R-Squared	0.9668					
Adj R-Squared	0.9027					

$$Y1=-24.84850+0.20836\times A+2.43025\times F+6.51175\times H-7.275E^{-3}\times AF+0.0181\times AH+0.0985\times FH-1.26E^{-3}\times A^2-0.13825\times F^2-0.99\times H^2$$

Supplemental material Table S5 The physicochemical indexes of 11 vegetable oils

Indexes[a]	soybean oil	rapeseed oil	camellia seed oil	sunflower oil	olive oil	walnut oil	peanut oil	rice oil	flaxseed oil	corn oil	sesame oil
C16:0, PA/%	10.52±0.10	4.51±0.05	7.32±0.08	5.47±0.06	9.22±0.11	5.3±0.06	11.71±0.12	18.12±0.18	6.08±0.07	12.34±0.16	8.74±0.11
C18:0, SA/%	3.83±0.04	2.58±0.03	2.24±0.02	5.42±0.07	3.43±0.04	2.21±0.02	3.4±0.04	1.68±0.018	3.24±0.05	2.01±0.02	5.62±0.07
C18:1,OA/%	24.4±0.29	59.11±0.76	80.82±1.13	23.21±0.32	79.61±1.14	17.67±0.24	41.86±0.54	40.22±0.48	19.16±0.21	29.16±0.32	39.04±0.56
C18:2,LA/%	52.16±0.67	20.68±0.29	8.26±0.12	63.56±0.95	4.79±0.071	65.1±0.97	36.88±0.51	31.02±0.43	17.25±0.20	54.87±0.65	45.62±0.79
C18:3,LNA/%	6.72±0.09	6.68±0.11	0.41±0.07	0.18±0.02	0.68±0.01	7.28±0.11	0.62±0.09	0.65±0.09	53.34±0.69	0.72±0.09	0.38±0.04
OFA/%	1.92±0.02	6.44±0.10	0.59±0.07	2.16±0.02	2.27±0.07	2.44±0.02	5.53±0.08	8.31±0.12	0.93±0.01	0.96±0.01	0.6±0.07
SFA/%	15.37±0.24	13.11±0.15	9.81±0.18	12.89±0.16	14.68±0.19	9.51±0.12	16.34±0.19	22.86±0.36	9.54±0.14	15±0.22	14.75±0.20
MFA/%	25.52±0.30	59.51±0.77	81.08±1.35	23.81±0.33	80.61±1.12	18.29±0.25	43.09±0.56	41.72±0.5	19.56±0.31	29.52±0.47	39.35±0.43
MFA/SFA	1.66±0.02	4.54±0.06	8.26±0.91	1.84±0.02	5.49±0.06	1.92±0.02	2.63±0.03	1.82±0.02	2.05±0.02	1.96±0.02	2.66±0.03
MFA/PFA	0.45±0.06	2.18±0.03	8.99±0.08	0.37±0.04	14.73±0.17	0.25±0.00	1.14±0.01	1.31±0.01	0.27±0.01	0.53±0.06	0.87±0.01
MFA/OFA	13.29±0.14	9.24±0.11	137.42±1.86	11.02±0.14	35.51±0.46	7.49±0.09	7.79±0.09	5.02±0.05	21.03±0.23	30.75±0.33	65.58±0.91
AV/(mg/g)	3.25±0.03	3.51±0.04	3.06±0.03	3.67±0.05	2.49±0.03	3.25±0.04	3.36±0.04	5.48±0.06	3.81±0.04	3.17±0.038	3.61±0.05
PV/(mmol/kg）	6.7±0.08	5.88±0.08	5.38±0.01	6.69±0.1	5.1±0.07	6.9±0.10	5.4±0.07	7.01±0.09	5.46±0.05	6.09±0.06	5.2±0.05
RI/n^{40}	1.47±0.02	1.47±0.01	1.46±0.03	1.46±0.02	1.47±0.023	1.47±0.02	1.46±0.01	1.46±0.02	1.48±0.016	1.46±0.016	1.46±0.01
IV/(g/100g)	133.28±1.99	110.16±0.32	96.28±1.59	136.22±1.49	94.43±1.08	156.48±1.72	90.17±1.08	108.42±1.62	194.36±2.32	123.46±1.48	117.51±1.52
SV/（mg/g）	192.79±3.08	174.51±2.26	194.46±2.33	191.11±2.29	190.35±2.24	186.72±2.24	189.21±2.45	186.61±2.98	192.42±2.50	189.5±2.46	191.02±2.67
UA/（mg/g）	10.91±0.13	12.8±0.17	8.41±0.19	9.15±0.11	8.12±0.10	13.8±0.17	5.72±0.08	7.56±0.09	13.12±0.18	18.1±0.25	15.31±0.22
Price/(yuan/L)	8±0.10	16.01±0.24	70.01±0.98	14±0.19	120.1±1.68	300±4.2	26±0.39	20±0.26	75±1.12	18±0.27	53±0.84
MAV/(%)	0.02±0.001	0.02±0.02	0.01±0.01	0.01±0	0.01±0	0.01±0.01	0.012±0	0.012±0.01	0.01±0.01	0.01±0.02	0.017±0.04
IT/(h)	2.11±0.025	1.99±0.021	6.75±0.11	2.24±0.03	8.85±0.13	1.15±0.01	1.92±0.02	4.06±0.04	0.83±0.09	4.65±0.05	5.07±0.06

[a]C16:0, PA-Palmitic acid; C18:0, SA-Stearic acid; C18:1,OA-Oleic acid; C18:2,LA-Linoleic acid; C18:3,LNA-Linolenic acid; OFA-Other fatty acids; SFA-Saturated fatty acid; MFA-Monounsaturated fatty acids; MFA/SFA-Monounsaturated fatty acids/saturated fatty acids; MFA/PFA-Monounsaturated fatty acids / polyunsaturated fatty acids; MFA/OFA-Monounsaturated fatty acids/other fatty acids; AV-Acid value; PV-Peroxide value; RI-Refractive index; IV-Iodine value;SV-Saponification value; UA-Unsaponifiable; Price (CNY);

MAV-Moisture and volatiles; IT-Induction time

Supplemental material Table S6 The correlate matrix between the factors[a]

	x1	x2	x3	x4	x5	x6	x7	x8	x9	x10	x11	x12	x13	x14	x15	x16	x17	x18	x19	x20
x1	1(0.000***)	-0.318(0.340)	0.004(0.990)	0.000(0.999)	-0.312(0.350)	0.445(0.171)	0.888(0.000***)	0.018(0.959)	-0.256(0.448)	-0.044(0.898)	-0.145(0.671)	0.536(0.089*)	0.217(0.521)	-0.39(0.236)	-0.392(0.233)	0.153(0.654)	-0.248(0.462)	-0.365(0.269)	0.448(0.166)	0.251(0.457)
x2	-0.318(0.340)	1(0.000***)	-0.193(0.569)	0.279(0.407)	-0.043(0.900)	-0.375(0.255)	-0.099(0.773)	-0.196(0.563)	-0.209(0.537)	-0.119(0.728)	-0.003(0.993)	-0.173(0.611)	-0.227(0.503)	0.013(0.970)	0.084(0.806)	0.305(0.362)	-0.001(0.997)	-0.229(0.499)	0.241(0.475)	-0.036(0.917)
x3	0.004(0.990)	-0.193(0.569)	1(0.000***)	-0.785(0.004***)	-0.371(0.261)	0.094(0.782)	0.028(0.935)	1(0.000***)	0.915(0.000***)	0.868(0.001***)	0.598(0.052*)	-0.323(0.333)	-0.577(0.063*)	-0.498(0.119)	-0.737(0.010***)	-0.1(0.769)	-0.413(0.207)	-0.095(0.782)	-0.265(0.431)	0.779(0.005***)
x4	0.000(0.999)	0.279(0.407)	-0.785(0.004***)	1(0.000***)	-0.244(0.469)	-0.117(0.731)	0.045(0.896)	-0.785(0.004***)	-0.734(0.010**)	-0.713(0.014**)	-0.429(0.188)	0.1(0.771)	0.65(0.030**)	-0.002(0.996)	0.299(0.372)	0.05(0.884)	0.381(0.248)	0.131(0.702)	0.475(0.140)	-0.507(0.112)
x5	-0.312(0.350)	-0.043(0.900)	-0.371(0.261)	-0.244(0.469)	1(0.000***)	-0.223(0.509)	-0.433(0.184)	-0.376(0.254)	-0.21(0.536)	-0.232(0.493)	-0.156(0.646)	0.107(0.754)	-0.156(0.648)	0.894(0.000***)	0.814(0.002***)	0.119(0.727)	0.218(0.519)	0.108(0.752)	-0.47(0.145)	-0.458(0.156)

x6	0.445(0.171)	-0.375(0.255)	0.094(0.782)	-0.117(0.731)	-0.223(0.509)	1(0.000***)	0.662(0.026**)	0.107(0.755)	-0.151(0.657)	-0.141(0.680)	-0.524(0.098*)	0.615(0.044**)	0.344(0.300)	-0.395(0.230)	-0.364(0.271)	-0.665(0.026**)	-0.465(0.150)	-0.215(0.526)	0.365(0.269)	-0.219(0.518)
x7	0.888(0.000***)	-0.099(0.773)	0.028(0.935)	0.045(0.896)	-0.433(0.184)	0.662(0.026**)	1(0.000***)	0.042(0.902)	-0.32(0.337)	-0.088(0.797)	-0.352(0.288)	0.606(0.048**)	0.275(0.413)	-0.513(0.107)	-0.485(0.130)	-0.145(0.670)	-0.312(0.349)	-0.491(0.125)	0.678(0.022**)	0.174(0.609)
x8	0.018(0.959)	-0.196(0.563)	1(0.000***)	-0.785(0.004***)	-0.376(0.254)	0.107(0.755)	0.042(0.902)	1(0.000***)	0.91(0.000***)	0.869(0.001***)	0.589(0.057*)	-0.315(0.345)	-0.57(0.067*)	-0.504(0.114)	-0.743(0.009***)	-0.1(0.769)	-0.426(0.192)	-0.097(0.776)	-0.258(0.444)	0.779(0.005***)
x9	-0.256(0.448)	-0.209(0.537)	0.915(0.000***)	-0.734(0.010**)	-0.21(0.536)	-0.151(0.657)	-0.32(0.337)	0.91(0.000***)	1(0.000***)	0.793(0.004***)	0.782(0.004***)	-0.433(0.184)	-0.567(0.069*)	-0.331(0.320)	-0.52(0.101)	0.027(0.936)	-0.303(0.365)	0.044(0.897)	-0.464(0.151)	0.651(0.030**)
x10	-0.044(0.898)	-0.119(0.728)	0.868(0.001***)	-0.713(0.014**)	-0.232(0.493)	-0.141(0.680)	-0.088(0.797)	0.869(0.001***)	0.793(0.004***)	1(0.000***)	0.493(0.123)	-0.489(0.127)	-0.526(0.097*)	-0.245(0.467)	-0.509(0.110)	0.161(0.637)	-0.396(0.228)	0.154(0.652)	-0.41(0.210)	0.844(0.001***)
x11	-0.145(0.671)	-0.003(0.993)	0.598(0.052*)	-0.429(0.188)	-0.156(0.646)	-0.524(0.098*)	-0.352(0.288)	0.589(0.057*)	0.782(0.004***)	0.493(0.123)	1(0.000***)	-0.315(0.346)	-0.519(0.102)	-0.202(0.551)	-0.303(0.365)	0.444(0.171)	-0.012(0.973)	-0.002(0.995)	-0.324(0.331)	0.61(0.046**)
x1	0.536(0.08	-0.173(0	-0.323(0.3	0.1(0.771)	0.107(0.75	0.615(0.04	0.606(0.04	-0.315(0.3	-0.433(0.1	-0.489(0.1	-0.315(0.3	1(0.000**	0.496(0.1	-0.021(0.9	0.127(0.70	-0.189(0.5	-0.151(0	-0.282(0	0.475(0.1	-0.313(0.3

2	9*)	.611)	33)		4)	4**)	8**)	45)	84)	27)	46)	*)	21)	52)	9)	77)	.658)	.401)	40)	49)
x13	0.217(0.521)	-0.227(0.503)	-0.577(0.063*)	0.65(0.030**)	-0.156(0.648)	0.344(0.300)	0.275(0.413)	-0.57(0.067*)	-0.567(0.069*)	-0.526(0.097*)	-0.519(0.102)	0.496(0.121)	1(0.000***)	-0.036(0.916)	0.273(0.417)	-0.174(0.610)	0.034(0.921)	0.109(0.749)	0.534(0.091*)	-0.476(0.138)
x14	-0.39(0.236)	0.013(0.970)	-0.498(0.119)	-0.002(0.996)	0.894(0.000***)	-0.395(0.230)	-0.513(0.107)	-0.504(0.114)	-0.331(0.320)	-0.245(0.467)	-0.202(0.551)	-0.021(0.952)	-0.036(0.916)	1(0.000***)	0.912(0.000***)	0.128(0.708)	0.476(0.139)	0.409(0.211)	-0.376(0.255)	-0.407(0.215)
x15	-0.392(0.233)	0.084(0.806)	-0.737(0.010***)	0.299(0.372)	0.814(0.002***)	-0.364(0.271)	-0.485(0.130)	-0.743(0.009***)	-0.52(0.101)	-0.509(0.110)	-0.303(0.365)	0.127(0.709)	0.273(0.417)	0.912(0.000***)	1(0.000***)	0.153(0.653)	0.473(0.142)	0.313(0.348)	-0.209(0.537)	-0.622(0.041**)
x16	0.153(0.654)	0.305(0.362)	-0.1(0.769)	0.05(0.884)	0.119(0.727)	-0.665(0.026**)	-0.145(0.670)	-0.1(0.769)	0.027(0.936)	0.161(0.637)	0.444(0.171)	-0.189(0.577)	-0.174(0.610)	0.128(0.708)	0.153(0.653)	1(0.000***)	-0.139(0.684)	0.008(0.980)	-0.214(0.527)	0.275(0.414)
x17	-0.248(0.462)	-0.001(0.997)	-0.413(0.207)	0.381(0.248)	0.218(0.519)	-0.465(0.150)	-0.312(0.349)	-0.426(0.192)	-0.303(0.365)	-0.396(0.228)	-0.012(0.973)	-0.151(0.658)	0.034(0.921)	0.476(0.139)	0.473(0.142)	-0.139(0.684)	1(0.000***)	0.154(0.652)	0.076(0.824)	-0.159(0.641)
x18	-0.365(0.269)	-0.229(0.499)	-0.095(0.782)	0.131(0.702)	0.108(0.752)	-0.215(0.526)	-0.491(0.125)	-0.097(0.776)	0.044(0.897)	0.154(0.652)	-0.002(0.995)	-0.282(0.401)	0.109(0.749)	0.409(0.211)	0.313(0.348)	0.008(0.980)	0.154(0.652)	1(0.000***)	-0.387(0.239)	-0.041(0.905)
x	0.448	0.24	-0.26	0.475	-0.47	0.365	0.678	-0.25	-0.46	-0.41	-0.32	0.47	0.53	-0.37	-0.20	-0.21	0.07	-0.3	1(0.0	-0.10

19	(0.166)	1(0.475)	5(0.431)	(0.140)	(0.145)	(0.269)	(0.022**)	8(0.444)	4(0.151)	(0.210)	4(0.331)	5(0.140)	4(0.091*)	6(0.255)	9(0.537)	4(0.527)	6(0.824)	87(0.239)	00***)	5(0.758)
x20	0.251 (0.457)	-0.036(0.917)	0.779 (0.005***)	-0.507(0.112)	-0.458(0.156)	-0.219(0.518)	0.174 (0.609)	0.779 (0.005***)	0.651 (0.030**)	0.844 (0.001***)	0.61(0.046**)	-0.313(0.349)	-0.476(0.138)	-0.407(0.215)	-0.622(0.041**)	0.275 (0.414)	-0.159(0.641)	-0.041(0.905)	-0.105(0.758)	1(0.000***)

[a]X1-Palmitic acid, X2-Stearic acid, X3-Oleic acid, X4-Linoleic acid, X5-Linolenic acid, X6-Other fatty acids, X7-Saturated fatty acid, X8-Monounsaturated fatty acids, X9-Monounsaturated fatty acids/saturated fatty acids, X10-Monounsaturated fatty acids / polyunsaturated fatty acids, X11-Monounsaturated fatty acids/other fatty acids, X12-Acid value, X13-Peroxide value, X14-Refractive index, X15-Iodine value, X16-Saponification number, X17-Unsaponifiable, X18-Price (CNY), X19-Moisture and volatiles, X20-Induction time

Supplemental material Table S7 Total variance interpretation

Component	Feature root	Variance contribution/%	Cumulative variance contribution/%
1	7.088	35.442	35.442
2	4.998	24.988	60.43
3	2.206	11.028	71.458
4	1.63	8.15	79.609
5	1.268	6.339	85.948
6	1.011	5.057	91.005
7	0.757	3.786	94.791
8	0.552	2.76	97.551

9	0.343	1.716	99.267
10	0.147	0.733	100
11		0	100
12		0	100
13		0	100
14		0	100
15		0	100
16		0	100
17		0	100
18		0	100
19		0	100
20		0	100

Supplemental material Table S8 Factor load factor

	PC1	PC2	PC3	PC4	PC5	Co-commonality (common factor variance)
x1	0.008	0.748	0.024	0.469	0.391	0.933
x2	-0.125	-0.152	0.657	0.119	-0.585	0.828
x3	0.975	0.09	-0.144	-0.069	-0.067	0.988
x4	-0.739	0.126	0.521	-0.345	0.157	0.977
x5	-0.378	-0.615	-0.479	0.467	-0.148	0.991
x6	-0.07	0.776	-0.576	-0.127	-0.142	0.976
x7	-0.035	0.923	0.016	0.287	0.06	0.94
x8	0.974	0.103	-0.147	-0.065	-0.064	0.989
x9	0.921	-0.209	-0.121	-0.116	-0.011	0.92
x10	0.895	-0.13	-0.069	0.015	0.148	0.845
x11	0.689	-0.324	0.291	0.148	0.158	0.712
x12	-0.425	0.579	-0.314	0.373	0.049	0.756
x13	-0.641	0.406	-0.028	-0.258	0.369	0.78
x14	-0.491	-0.715	-0.305	0.252	0.063	0.912
x15	-0.724	-0.606	-0.17	0.185	0.06	0.958
x16	0.097	-0.321	0.549	0.6	0.338	0.889
x17	-0.41	-0.393	0.195	-0.202	0.103	0.412
x18	-0.071	-0.476	-0.186	-0.433	0.545	0.75

x19	-0.345	0.716	0.362	-0.072	-0.093	0.776
x20	0.834	0.08	0.259	0.134	0.264	0.857

Supplemental material Table S9 Feature vector standardization

	PC1	PC2	PC3	PC4	PC5
x1	0.001	0.15	0.011	0.288	0.308
x2	-0.018	-0.03	0.298	0.073	-0.462
x3	0.138	0.018	-0.065	-0.043	-0.053
x4	-0.104	0.025	0.236	-0.212	0.124
x5	-0.053	-0.123	-0.217	0.287	-0.117
x6	-0.01	0.155	-0.261	-0.078	-0.112
x7	-0.005	0.185	0.007	0.176	0.047
x8	0.137	0.021	-0.067	-0.04	-0.05
x9	0.13	-0.042	-0.055	-0.071	-0.009
x10	0.126	-0.026	-0.031	0.009	0.117
x11	0.097	-0.065	0.132	0.091	0.124
x12	-0.06	0.116	-0.142	0.229	0.039
x13	-0.09	0.081	-0.013	-0.158	0.291
x14	-0.069	-0.143	-0.138	0.155	0.05
x15	-0.102	-0.121	-0.077	0.114	0.048
x16	0.014	-0.064	0.249	0.368	0.267
x17	-0.058	-0.079	0.088	-0.124	0.081
x18	-0.01	-0.095	-0.084	-0.266	0.43
x19	-0.049	0.143	0.164	-0.044	-0.073
x20	0.118	0.016	0.117	0.082	0.209

Supplemental material Table S10 Principal components and overall scores

Rank	Vegetable oils	Composite score	PC1	PC2	PC3	PC4	PC5
1	Camellia seed oil	0.683	1.925	-0.673	0.153	0.158	0.678
2	Olive oil	0.65	1.74	-0.262	-0.142	0.06	0.288
3	Rice oil	0.529	-0.337	2.169	-1.026	1.006	1.001
4	Peanut oil	0.229	0.181	0.738	-0.148	0.151	-0.749
5	Sesame oil	0.138	-0.009	-0.132	1.569	0.334	-0.718
6	Corn oil	0.02	-0.354	0.052	0.689	-0.102	0.981
7	Soybean oil	-0.053	-0.706	0.328	0.91	0.281	-0.017
8	Sunflower oil	-0.214	-0.696	0.083	1.125	-0.314	-0.896
9	Rapeseed oil	-0.255	0.233	0.355	-1.416	-1.556	-1.702
10	Walnut oil	-0.785	-1.044	-0.858	-0.422	-1.772	1.585
11	Flaxseed oil	-0.94	-0.933	-1.798	-1.292	1.755	-0.453

Supplemental material Table S11 Experimental design and results of Box-Benhnken in CSO deacidification

NO.	A-Deacidification temperature (℃)[a]	B-Reaction time (min)[b]	C-Excess alkali amount (%)[c]	Acid value (mgKOH/g)
1	-1	-1	0	0.142
2	-1	1	0	0.128
3	0	1	-1	0.125
4	0	-1	1	0.110
5	-1	0	-1	0.116
6	-1	0	1	0.108
7	0	0	0	0.056
8	0	0	0	0.064
9	1	-1	0	0.134
10	1	0	-1	0.130
11	0	-1	-1	0.120
12	0	1	1	0.106
13	1	0	1	0.108
14	0	0	0	0.070
15	1	1	0	0.098

[a]Deacidification temperature was set to 80℃ (-1), 85℃(0) and 90℃ (1) ;

[b]Reaction time was set to 55min (-1), 65min (0) and 75min (1);

[c]Excess alkali amount was set to 0.1%(-1), 0.2%(0) and 0.3%(1).

Supplemental material Table S12 Variance analysis of parameters in regression equation of integrated membership grade in CSO deacidification

Source	Sum of squares	df	Mean square	F-value	p-value	Significance
Model	8.40E-03	9	9.33E-04	7.13	0.0218	*
A-A	1.45E-04	1	1.45E-05	1.1	0.3415	
B-B	3.00E-04	1	3.00E-04	2.29	0.1904	
C-C	3.00E-04	1	3.00E-04	2.29	0.1904	
AB	1.21E-04	1	1.21E-04	0.92	0.3805	
AC	4.00E-05	1	4.01E-05	0.031	0.8681	
BC	2.03E-05	1	2.03E-05	0.15	0.7103	
A^2	3.31E-03	1	3.31E-03	25.32	0.0040	**
B^2	3.83E-03	1	3.83E-03	29.27	0.0029	**
C^2	1.40E-04	1	1.40E-04	10.96	0.0212	**
Residual	6.54E-03	5	1.43E-04			
Lack of Fit	5.56E-04	3	1.31E-04	3.76	0.2174	not significant
Pure Error	9.95E-04	2	1.85E-04			
Cor Total	9.05E-03	14				
R-Squared	0.9734					
Adj R-Squared	0.9420					

$$Y2=2.30283-0.16388\times A-0.035288\times B-2.09750\times C-11.4753\times AB-0.010\times AC-15.2323\times BC+17.3588\times A^2+ 4.7551\times B^2+7.2883\times C^2$$

Supplemental material Table S13 Experimental design and results of Box-Benhnken in CSO decolorization

NO.	Decolorization temperature (°C)[a]	Decolorization time (min)[b]	Decolorizer dosage (%)[c]	Decolorization rate (%)
1	-1	-1	0	82.13
2	-1	1	0	84.67
3	0	1	-1	89.04
4	0	-1	1	90.89
5	-1	0	-1	83.42
6	-1	0	1	87.59
7	0	0	0	88.71
8	0	0	0	91.05
9	1	-1	0	83.46
10	1	0	-1	88.26
11	0	-1	-1	87.18
12	0	1	1	92.48
13	1	0	1	92.52
14	0	0	0	91.79
15	1	1	0	92.03

[a]Decolorization temperature was set to 75°C (-1), 85°C(0) and 95°C (1) ;

[b]Decolorization time was set to 15min (-1), 25min (0) and 35min (1);

[c]Decolorizer dosage was set to 2%(-1), 3%(0) and 4%(1).

Supplemental material Table S14 Variance analysis of parameters in regression equation of integrated membership grade in CSO decolorization

Source	Sum of Squares	df	Mean Square	F-value	Prob > F	Significance
Model	174.5	9	19.39	46.87	0.0003	**
A-A	14.85	1	14.85	35.9	0.0019	**
B-B	67.45	1	67.45	163.07	< 0.0001	**
C-C	34.82	1	34.82	84.17	0.0003	**
AB	0.12	1	0.12	0.29	0.6147	
AC	8.40E-01	1	8.40E-01	2.02E+00	0.2141	
BC	0.063	1	0.063	0.15	0.7135	
A^2	28.78	1	28.78	69.56	0.0004	**
B^2	25.72	1	25.72	62.17	0.0005	**
C^2	9.8	1	9.8	23.69	0.0046	**
Residual	2.07	5	0.41			
Lack of Fit	1.79	3	0.6	4.31	0.1939	
Pure Error	0.28	2	0.14			
Cor Total	176.57	14				
R-Squared	0.9883					
Adj R-Squared	0.9672					

$Y3=-161.340+4.722\times A+1.948\times B+14.884\times C-7.689\times AB-0.046\times AC+0.013\times BC-0.027\times A^2-0.026\times B^2-1.586\times C^2$

Supplemental material Table S15 The fatty acid composition and conventional physicochemical indexes of CSO and other pharmacopoeias

Parameters	This study-injection CSO	Chinese Pharmacopoeia	United States Pharmacopoeia	European Pharmacopoeia	Japanese Pharmacopoeia
Characters	Clarification,transparency, odorless	Pale yellow transparent liquid; Odorless or almost odorless	Transparent,almost transparent liquid	Pale yellow transparent liquid; Odorless or almost odorless	Pale yellow transparent liquid; Odorless or almost odorless
Relative density	0.916	0.916～0.922	0.916～0.922	0.910-0.930	0.920-0.938
Refractive index（20 ℃）	1.473	1.472～1.476	1.460-1.70	1.470-0.476	1.465-1.475
Acid value	0.0515（mgKOH/g）	≤0.1（mgKOH/g）	≤0.6（mgNaOH/g）(pH6-8)	≤0.5（mg KOH/g）	≤0.5（mgNaOH/g）(pH6-8)
Saponification value	187（mgKOH/g）	188～195（mgKOH/g）	189-195（mgKOH/g）	0.1-0.2（gNaOH/g）	189-195（mgKOH/g）
Iodine value	72（g/100g）	126～140（g/100g）	0.4-0.7（g/100g）	50（g/100g）	50-111（g/100g）
Absorbance	0.015	≤0.045	-	-	-
Peroxide value（%)	0.82	≤3ml	5 mg/L	0.2mg/g	0.25mg/g
Unsaponifiable（%）	0.51%	≤1.0%	≤1%	≤0.05%	≤0.15%
Moisture and volatiles（%）	0.03%	≤0.1%	0.2%	0.1-0.2%	0.05%

Fatty acid composition	Palmitic acid 7.12%, stearic acid 2.03%, oleic acid 81.09%, linoleic acid 9.12%, linolenic acid 0.28%, unsaturated fat 90.49%	Palmitic acid 7.0%~14.0%, stearic acid 1.0%~6.0%, oleic acid 18.0%~30.0%, linoleic acid 44.0%~62.0%, linolenic acid 4.0%~11.0%	Palmitic acid ~10%, stearic acid -0.3%, oleic acid 98%~107%, linoleic acid 55.0%~75.0%, linolenic acid -30.0%	Palmitic acid ~10%, stearic acid -05%, oleic acid 75%~85%, linoleic acid 50.0%~75.0%, linolenic acid -50%	Palmitic acid ~2%, stearic acid -2%, oleic acid 98%, linoleic acid 50.0%, linolenic acid -50%
Heavy metal limits（mg/kg）	1	≤2	≤0.5	≤5	≤1.1ppm
